# BDNF Depresses Excitability of Parvalbumin-Positive Interneurons through an M-Like Current in Rat Dentate Gyrus

**DOI:** 10.1371/journal.pone.0067318

**Published:** 2013-06-19

**Authors:** Jose Luis Nieto-Gonzalez, Kimmo Jensen

**Affiliations:** 1 Department of Biomedicine, Aarhus University, Aarhus C, Denmark; 2 Lundbeck Foundation Research Center MIND, Aarhus University, Aarhus C, Denmark; University of Houston, United States of America

## Abstract

In addition to their classical roles in neuronal growth, survival and differentiation, neurotrophins are also rapid regulators of excitability, synaptic transmission and activity-dependent synaptic plasticity. We have recently shown that mature BDNF (Brain Derived Neurotrophic Factor), but not proBDNF, modulates the excitability of interneurons in dentate gyrus within minutes. Here, we used brain slice patch-clamp recordings to study the mechanisms through which BDNF modulates the firing of interneurons in rat dentate gyrus by binding to TrkB receptors. Bath application of BDNF (15 ng/ml) under current-clamp decreased the firing frequency (by 80%) and input resistance, blocking the delayed firing observed at near-threshold voltage ranges, with no changes in resting membrane potential or action potential waveform. Using TEA (tetraethylammonium), or XE991(a Kv7/KCNQ channel antagonist), the effect of BDNF was abolished, whereas application of retigabine (a Kv7/KCNQ channel opener) mimicked the effect of BDNF, suggesting that the M-current could be implicated in the modulation of the firing. In voltage-clamp experiments, BDNF increased the M-like current amplitude with no change in holding current. This effect was again blocked by XE991 and mimicked by retigabine, the latter accompanied with a change in holding current. In agreement with the electrophysiology, parvalbumin-positive interneurons co-expressed TrkB receptors and Kv7.2/KCNQ2 channels. In conclusion, BDNF depresses the excitability of interneurons by activating an M-like current and possibly blocking Kv1 channels, thereby controlling interneuron resting membrane potential and excitability.

## Introduction

Brain-derived neurotrophic factor (BDNF) is an important neurotrophin involved in the regulation of neuron survival and differentiation during development [1]. Moreover, the differentiation and maturation of progenitor cells is promoted by local BDNF being synthesized in dendrites of principal neurons [2]. The importance of BDNF is exemplified by the poor viability of mice that are null-mutants for BDNF or the corresponding neurotrophin receptors. Interestingly, BDNF also has rapid cellular actions, including effects on neuronal excitability, synaptic transmission, and plasticity [1], [3], [4]. For this to occur, BDNF acts by binding to two types of plasma membrane receptors, the Trk receptor tyrosine kinase B (TrkB) or the p75 pan-neurotrophin receptor (p75NTR;[5]). BDNF binding to TrkB receptors triggers receptor dimerization and phosphorylation of tyrosine residues, and, interestingly, tyrosine phosphorylation can produce rapid changes in ion channel function [4], [6]. Recently, we described that mature BDNF, but not proBDNF, depresses the excitability of fast-spiking parvalbumin-positive interneurons within minutes [7]. This effect was mediated by activation of TrkB, and not p75NTR, and led to a decreased firing upon interneuron depolarization with little or no change in other electrophysiological parameters. Thus, it can be hypothesized that BDNF-mediated TrkB activation may lead to a rapid modulation of ion channels in the interneuron membrane.

Among possible mechanisms that could contribute to regulation of interneuron excitability by BDNF on a rapid time scale is activation of an M-current. The modulation of M-currents has profound effects on neuronal excitability, being a common target of a variety of transmitters and hormones acting on G protein-coupled receptors, and it was previously reported that M-currents control interspike intervals in hippocampal somatostatin-containing interneurons [8]. The M-current is a slowly activating voltage-regulated potassium current, which is active at subthreshold potentials and inactivates poorly, generating steady voltage-dependent outward currents, that assist the cell in stabilizing the membrane potential in the presence of depolarizing currents (for review see [9], [10]). Thus, its slow activation and deactivation is important for their function as a brake for repetitive action potential firing.

In this study, we investigated whether BDNF can modulate potassium channels capable of suppressing action potential firing. Our results indicate that TrkB activation Holm et al

HEre, Here causes potentiation of specific potassium channels important for interneuron excitability in the rat dentate gyrus.

## Materials and Methods

### Ethics Statement

The experiments were carried out using Wistar rats of both sexes. The animals were kept in a university animal facility with a 12/12-h light/dark cycle with unrestricted access to food and water, in strict accordance with Danish and European legislation regarding the welfare of laboratory animals. No experiments were performed on living animals. Procedures for housing and sacrificing of rats were approved by the Animal Welfare Officer at the Faculty of Health Sciences, Aarhus University.

### Brain-slice preparation

Rats (P14–P20) were deeply anesthetized with a mixture of O_2_ and 3.5% isoflurane until the tail-pinch reflex was absent. The rats were decapitated and brains were rapidly dissected out and transferred to ice-cold artificial cerebrospinal fluid (ACSF) composed of (in mM): 126 NaCl, 2.5 KCl, 2 CaCl_2_, 2 MgCl_2_, 1.25 NaH_2_PO_4_, 26 NaHCO_3_, 10 D-glucose (osmolality 305–315 mosmol⋅kg^−1^), pH 7.4 when bubbled with carbogen (5% CO_2_/95% O_2_). 350 µm thick coronal slices were cut on a Vibratome VT1200S (Leica) and stored for at least 1 h at room temperature before recording. To improve slice viability, kynurenic acid (3 mM), ascorbate (0.2 mM), and pyruvate (0.2 mM) were present during slicing and storage [11], which are glutamate receptors antagonists and antioxidants, respectively.

### Electrophysiological patch-clamp recordings from dentate gyrus interneurons

Slices were placed in a recording chamber at 33±1°C and perfused with bubbled ACSF at 2–3 ml⋅min^–1^. Neurons were visualized within the slice using a custom-built infrared videomicroscope equipped with a 40x water-immersion objective (Olympus, Denmark). Patch-pipettes (resistance 3–5 MΩ) were pulled from borosilicate glass (outer diameter  =  1.5 mm, inner diameter  =  0.8 mm from King Precision Glass Inc, Claremont, CA) on a DMZ Universal Puller (Zeitz Instruments, Munich, Germany) and filled with solution containing (in mM): 120 K-gluconate, 10 KCl, 10 phosphocreatine disodium salt, 2 MgATP, 0.3 NaGTP, 0.1 EGTA, 10 HEPES, pH 7.2 adjusted with KOH, osmolality 280–290 mosmol⋅kg^−1^. Modifications were done when BAPTA (1,2-Bis(2-Aminophenoxy)ethane-N,N,N′,N′-tetraacetic acid) or GDP-β-S (Guanosine 5′-[β-thio]diphosphate) were applied intracellularly. For BAPTA experiments, K-gluconate was reduced to 110 mM, while for GDP-β-S experiments, NaGTP was omitted. After obtaining stable gigaseals (>1 GΩ), electrophysiological recordings were made using a Multiclamp 700B amplifier. All interneurons that were included for analysis showed a stable resting membrane potential of at least –55 mV in the presence of kynurenic acid (2 mM), and action potentials that overshot 0 mV in current-clamp conditions. Under voltage-clamp conditions the extracellular solution contained: kynurenic acid (2 mM) to block ionotropic glutamate receptors, bicuculline (10 µM), a GABA_A_ receptor antagonist and tetrodotoxin (TTX, 1 µM) to block action potentials. Furthermore, the hyperpolarization-activated inward mixed cation current was blocked with ZD7288 (20 µM) and L-type Ca^2+^ channels with diltiazem (25 µM). For voltage-clamp experiments, series resistance and whole-cell capacitance were monitored and the recording discontinued if the series resistance increased by 50% or exceeded 20 MΩ. Stepping the membrane from –30 to –50 mV revealed an M-current (I_M_) through its distinct voltage dependence and time course of deactivation, and thereby distinguishing this current from other K^+^ channels.

### Analysis and data acquisition

Currents were low-pass filtered (eight-pole Bessel) at 3 kHz, digitized at 20 kHz, and acquired using a BNC-2110 DA converter and a PCI-6014 board (National Instruments, Austin, TX) combined with a custom-written LabVIEW 6.1–based software (EVAN v. 1.4, courtesy of Istvan Mody, UCLA). To calculate the amplitude of the I_M_, the deactivating current, obtained from an average of 10 sweeps, was fitted to a single exponential from which we obtained the amplitude [7].

In current-clamp recordings, the baseline membrane potential was measured over the 100–200 ms preceding the onset of the current injection. Input resistance was determined by injecting negative current pulses (500 ms, 1 Hz) estimating the slope of the voltage–current plot. Due to the presence of inward rectification or sag, the voltage value was obtained during the last 100 ms of the pulse. These plots showed a good linear fit (r>0.98; *P*<0.001). The average delay to first spike was measured from current

onset to the action potential peak. To quantify spike features, action potentials were detected using a first derivative function; spike onset was the value of the membrane potential at which the first derivative surpassed 10 V/s [12]. Paired t-tests were used for statistical comparisons of single neurons before and after application of a drug. For multiple comparisons, we first tested for significant differences using a one-way ANOVA test, and if so a Bonferroni test was used to perform pairwise comparisons between groups, and the significance level was *P*<0.05. Data are presented as means ± standard error of the mean, with n indicating the number of neurons.

### Identification of interneurons

Parvalbumin-positive interneurons in the dentate gyrus [13], [14] were identified after biocytin injection [7]. Intracellular labeling was performed by including 0.5% biocytin in the pipette solution and biocytin injection was facilitated by depolarizing current steps (0.2–1 nA) of 500 ms at 1 Hz for 10–20 min following data acquisition. Slices were transferred to paraformaldehyde (PFA) 4% overnight at 4°C and washed with PBS and incubated in a blocking solution comprising of 3% normal donkey serum and 0.3 Triton X-100. Slices were incubated in primary antibody goat anti-parvalbumin (Swant, 1∶500) in PBS containing 3% normal donkey serum and 0.3% Triton X-100 for 24 h at 4°C. Next, incubation with secondary antibody (Donkey anti-goat-Alexa 568, 1∶500, Invitrogen) with FITC-conjugated avidin-D in PBS and 0.3% Triton X-100 at 4°C overnight was performed. Finally, slices were embedded with fluorescent mounting medium and visualized in a confocal Zeiss LSM510 microscope.

### Immunohistochemistry for parvalbumin, KCNQ2 channels and TrkB receptors

After an overdose of sodium pentobarbital (50 mg/ml), animals (P15–P20) were transcardially perfused with 0.9% physiological saline followed by 4% PFA (pH 7.4). The brains were removed and postfixed overnight in the same solution at 4 °C. Brains were transferred to a solution consisting of 30% sucrose (diluted in phosphate buffer, pH 7.0–7.4) and stored at 4°C until they sank. Transversal sections of 40 µm were cut through the entire dentate gyrus along the rostrocaudal axis and stored in cryoprotectant antifreeze solution (50% Glycerol + 50% PBS). After washing with PBS, slices were blocked in blocking buffer (3% donkey serum with 0.3% Triton X-100 in PBS) at room temperature for 1 h. Then, slices were incubated in the same blocking solution with the following primary antibodies: rabbit anti-parvalbumin (SYSY, 1∶200), mouse anti-KCNQ2 (Abcam, 1∶500) and goat anti-mouse TrkB (R&D system, 1∶200) for 2 days at 4°C. After washing with PBS, slices were incubated with the secondary: donkey anti-rabbit conjugated with Alexa 647 (1∶500, invitrogen), donkey anti-mouse conjugated with Alexa 568 (1∶500, invitrogen) and donkey anti-goat conjugated with Dylight 488 (1∶500, Jackson Immunoresearch) antibodies for 2 days at 4°C. Finally, slices were mounted on slides and coverslipped with fluorescence mounting media (DAKO). Images were acquired on a Zeiss LSM510 microscope using 10x or 40x objectives.

### Drugs and solutions

All drugs and chemicals were from Sigma-Aldrich (St. Louis, MI), except for TTX (Alomone, Israel), kynurenic acid, diltiazem and ZD7288 (Tocris). BDNF was dissolved in ACSF in stock solutions at 5 µg/ml.

## Results

### Effect of BDNF on electrophysiological properties of parvalbumin-positive interneurons

**Figure 1:**
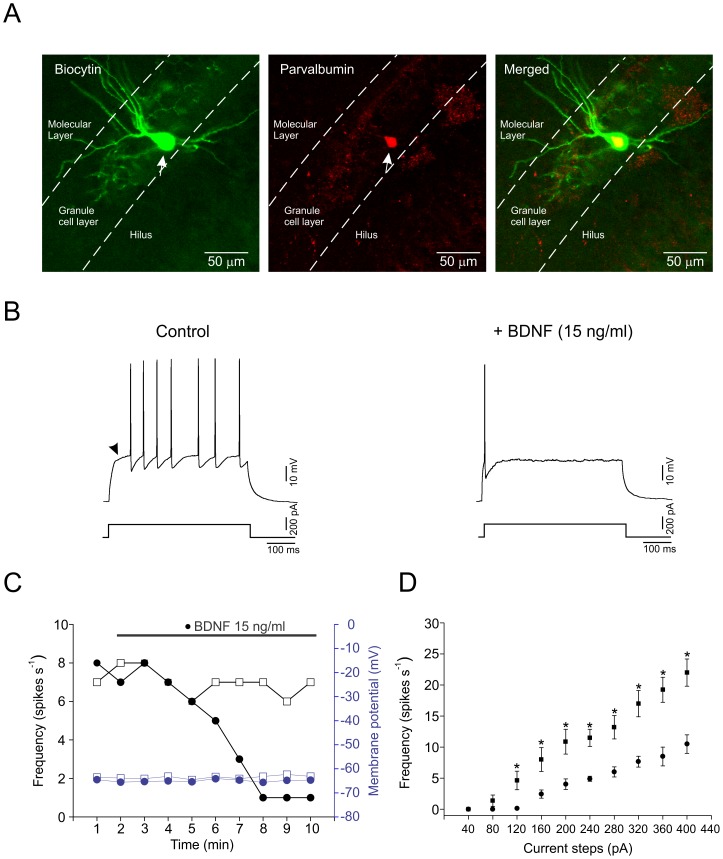
Identification of parvalbumin-positive interneurons and effect of BDNF on firing properties. A. Confocal image showing the co-localization of an intracellularly biocytin-labeled interneuron (green) and immunoreactivity for parvalbumin (red). B. Current-clamp recording from a parvalbumin-positive interneuron showing the firing before and after 10 min of bath perfusion of BDNF (15 ng/ml). The neuron was depolarized by a 200 pA current injection for 500 ms. Arrowhead indicates a delayed firing observed in the interneuron near the current threshold. C. The time course of the evoked firing (black y axis) and resting membrane potential (blue y axis) of an interneuron exposed to BDNF (black and blue circles) and a time-matched control (black and blue squares) exposed to control ACSF. The time of BDNF application is indicated by the solid line. D. Relationship between the firing frequency versus increasing current steps (0–400 pA) for control (squares) and after 10 min of BDNF exposure (circles) (n = 8).

Putative parvalbumin-containing interneurons were identified at the granule cells layer - hilar border of the dentate gyrus and somatic whole-cell recordings were carried out. A subset (n = 46) of the recorded interneurons (totaling n = 98) were injected with biocytin and immunostained against parvalbumin (Fig. 1A). Only 3 of the injected neurons were negative for parvalbumin indicating that our data were generally not skewed towards other neuronal subtypes not expressing parvalbumin. Bath application of BDNF (15 ng/ml) caused a decrease in their firing frequency (Fig. 1B,C). In current-clamp, interneurons (n = 12) showed a resting membrane potential of -63.0±3.3 mV with no significant difference when compared before and after bath application of BDNF (–65.3±2.9 mV). However, BDNF produced a significant decrease in input resistance (control  = 231.2±12.7 MΩ; BDNF  = 194.9±20.5 MΩ, n = 10, *P*<0.05). Also, when plotting the firing frequency against the injected current (40–400 pA), interneurons showed a smaller firing increase in presence of BDNF (Fig. 1D, *P*<0.05). Another firing characteristic of the interneurons was a delay to the first spike near current threshold (93.6±12.4 ms, n = 6), as marked by an arrowhead in Fig. 1B. This was frequent (> 80%) among the recorded parvalbumin-positive interneurons, similar to the data reported in fast-spiking cell types [15]. On the other hand, after bath application of BDNF, the delay to firing was clearly reduced (19.8±7.2 ms, n = 6, *P*<0.05). These data indicate that opening of ion channels, which depresses interneuron firing, could be potentiated. Possible candidate targets include potassium channels.

### BDNF does not change action potential waveform

**Figure 2:**
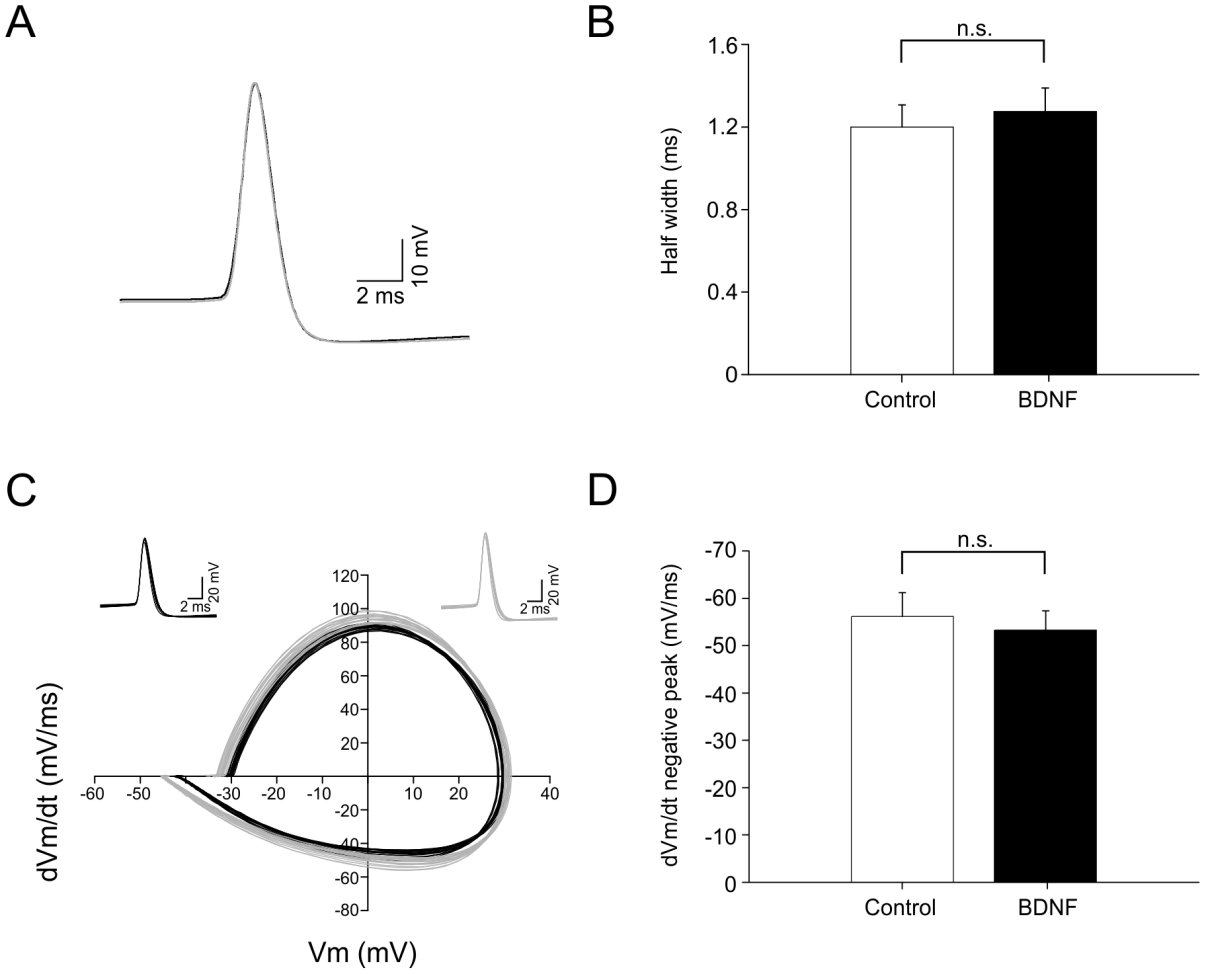
Action potential waveform is not affected by BDNF. A. Average action potential waveform before (black trace) and after (grey trace) bath application of BDNF (151ng/ml). B. Histogram showing no significant difference in the action potential half width in control and BDNF. C. Graph illustrating a representative phase plot of dV/dt versus voltage of 20 action potentials before (black lines) and after bath application of BDNF (grey lines). Insets illustrate the overlay of the 20 action potentials. D. Histogram showing no significant difference for dV/dt negative peak in both conditions control and exposed to BDNF (n = 8, *P*>0.05).

To investigate whether BDNF could change the action potential waveform, we analyzed the action potentials before and after BDNF application. Interneurons showed no significant change in the spike half-width (control 1.20±0.1 ms, n = 8) when exposed to BDNF (1.27±0.11 ms, n = 8, *P*>0.05, Fig. 2A,B). In addition, the peak of the negative dV/dt, a measure of action potential repolarization rate, did not show significant differences between control and BDNF (control  = –56.1±5.0 mV/ms, BDNF  = –53.2±4.0, n = 8, *P*>0.05, Fig. 2C,D).

### Potassium channel blockers abolish the effect of BDNF

**Figure 3:**
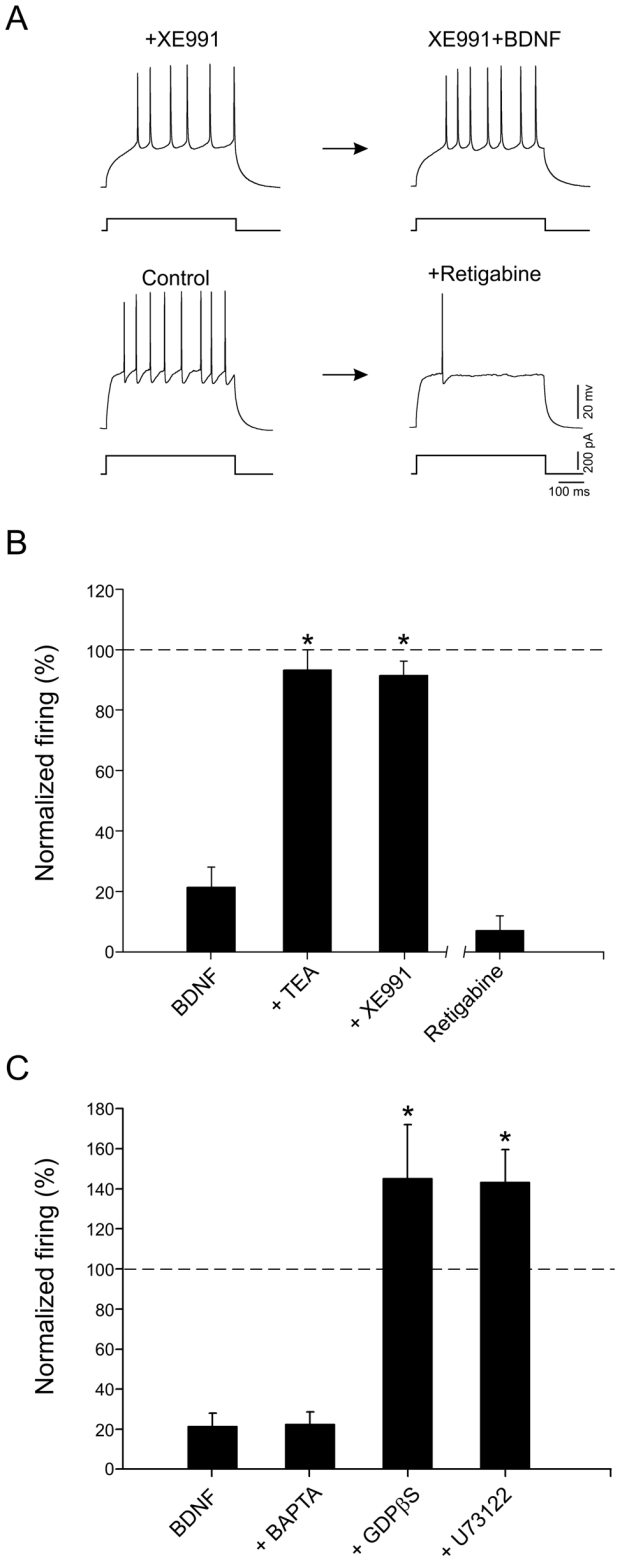
BDNF modulates M current. A. Current-clamp recordings showing evoked firing before and after bath application of BDNF (15 ng/ml) in presence of XE991 (10 µM), which blocked the neurotrophin effect. In agreement with the involvement of I_M_, the Kv7/KCNQ potentiator retigabine (10 µM) depressed interneuron firing. B. Histogram showing the effect of BDNF (15 ng/ml) in the presence of a broad spectrum potassium channels antagonist (TEA, 20 mM), the specific I_M_ blocker (XE991, 10 µM), or application of retigabine (10 µM) on the normalized firing of parvalbumin-positive interneurons. Note that XE991 blocked the effect of the BDNF while retigabine mimicked the effect of BDNF. C. Histogram depicting the effect of BDNF (15 ng/ml) on the normalized firing of interneurons recorded with an intracellular solution containing different inhibitors of intracellular cascades (BAPTA, 10 mM, GDP-β-S, 1 mM or U73122, 1 µM). The firing increased after application of BDNF in interneurons recorded with an intracellular G protein inhibitor (GDP-β-S) or a PLC inhibitor (U73122).

As shown above, BDNF produced a decrease in the firing frequency of dentate gyrus interneurons to 20.4±6.4% of the control level (Fig. 3B). To investigate possible potassium currents implicated in this modulation, we first applied the potassium channel blocker TEA. TEA (20 mM) fully blocked the effect of BDNF on firing frequency (TEA  = 93.3±6.5% of control, n = 10, *P*>0.05, Fig. 3B). Since potassium conductances generally regulate neuronal excitability [10] and Kv7/KCNQ/M channels specifically control interspike interval in interneurons [8], we investigated whether M-channels could be modulated by BDNF. After at least 5 min perfusion of the M-channel blocker XE991 (10 µM), interneurons were subsequently exposed to BDNF (15 ng/ml), now leading to no significant change in the delay to the first spike (XE991  = 62.5±9.9 ms; BDNF  = 59.4±11.8 ms, n = 8, *P*>0.05) and the firing frequency (91.4±4.8% of the control, n = 8, *P*>0.05, Fig. 3A,B). In agreement with the notion that BDNF activates an I_M_ on these interneurons, retigabine (10 µM), an agonist of M-channels mimicked the BDNF effect and decreased firing to 7±4.8% of control (n = 5, *P*>0.05, Fig. 3A,B), however no change was observed in the delay to the first spike (Retigabine  = 59.1±7.3 ms; BDNF  = 65.2±9.5 ms, n = 5, *P*>0.05). Altogether, these data indicate that Kv7/KCNQ/M channels are modulated by BDNF via TrkB receptors.

### The depressant effect on excitability of BDNF is mediated by G proteins and phospholipase C

To investigate the intracellular cascade through which BDNF could modulate the firing pattern of dentate gyrus interneurons, we included BAPTA (10 mM), GDB-β-S (1 mM) or U73122 (10 µM) in the patch-pipette solution to block intracellular calcium rises, G protein and phospholipase C (PLC) signaling, respectively. As summarized in Fig. 3C, BDNF reduced the firing significantly when BAPTA was present in the intracellular solution (to 19.8±4.4% of control), but not when GDB-β-S or U73122 was added to the intracellular solution, which even led to a BDNF-induced increase in firing (145.3±26.8% and 143.9±16.3% of control, *P*<0.05, respectively).

### BDNF modulates the amplitude of the I_M_

**Figure 4:**
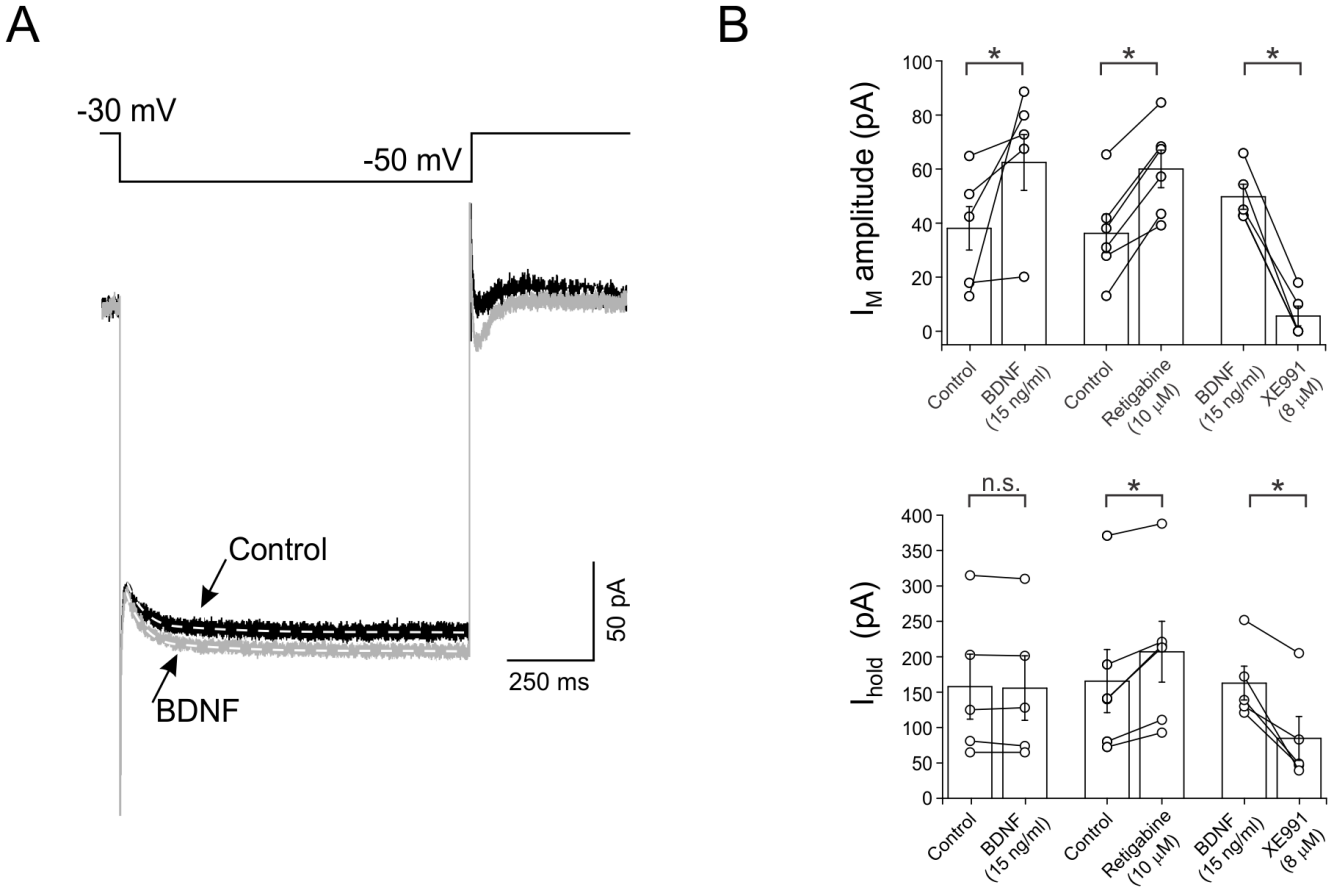
Voltage-clamp experiments reveal that an M-like current is potentiated by BDNF. A. Under voltage-clamp conditions stepping the membrane from –30 to –50 mV, BDNF (15 ng/ml) increased the I_M_ amplitude. Dashed lines indicate single exponential fits from which the I_M_ amplitude was obtained. B. Plots of I_M_ amplitude and holding current (I_hold_) are shown in different conditions. Again, retigabine mimicked the potentiating effect of BDNF on I_M_ amplitude.

Voltage-clamp experiments were carried out to isolate I_M_ and determine whether BDNF could modulate its amplitude. Interneurons were held at –30 mV and stepped to –50 mV (1 s, 0.1 Hz) revealing the deactivation of a slow outward current consistent with I_M_ (Fig. 4A). Only interneurons showing stable holding currents at –30 mV were included. A single exponential fit was adjusted to the deactivation phase of the current at –50 mV to obtain the amplitude of the I_M_ (Fig. 4A, dashed line). In control, interneurons showed an I_M_ amplitude of 38.1±8.0 pA (n = 6, Fig. 4B). After application of BDNF (15 ng/ml), we observed a significant increase in the I_M_ amplitude to 62.4±10.3 pA (n = 6, *P*<0.05) with no concurrent changes in holding current (control  = 149.2±38.5 pA, BDNF  = 143.2±39.2 pA, n = 6, *P*>0.05, Fig. 4B). Retigabine (10 µM) mimicked the effect of BDNF on the amplitude of I_M_ (control  = 36.3±7.1 pA, retigabine  = 60.1±6.9 pA, n = 6, *P*<0.05, Fig. 4B). Following retigabine, however, a significant increase in the holding current was observed (control  = 165.6±44.7 pA, retigabine  = 206.9±49.9 pA, n = 6, *P*<0.05, Fig. 4B). To block the effect of the BDNF on the amplitude of the I_M_, XE991 (10 µM) was applied to the bath after incubation of BDNF for at least 10 min. XE991 produced a significant decrease in the amplitude of the I_M_ (BDNF  = 49.7±4.6 pA, XE991  = 5.62±3.6 pA, n = 5, *P*<0.05, Fig. 4B) and also producing a decrease in the holding current (BDNF  = 162.8±23.9, XE991  = 84.6±31.01 pA, n5, *P*<0.05, Fig. 4B). These data point to a modulation of I_M_ by BDNF through TrkB receptors. However, another current, probably mediated by Kv1 channels (see Discussion), could also be implied counteracting the changes in holding current observed when retigabine, but not BDNF, was applied.

### TrkB receptors and Kv7.2/KCNQ2 channels co-localize in parvalbumin-positive interneurons in rat dentate gyrus

**Figure 5:**
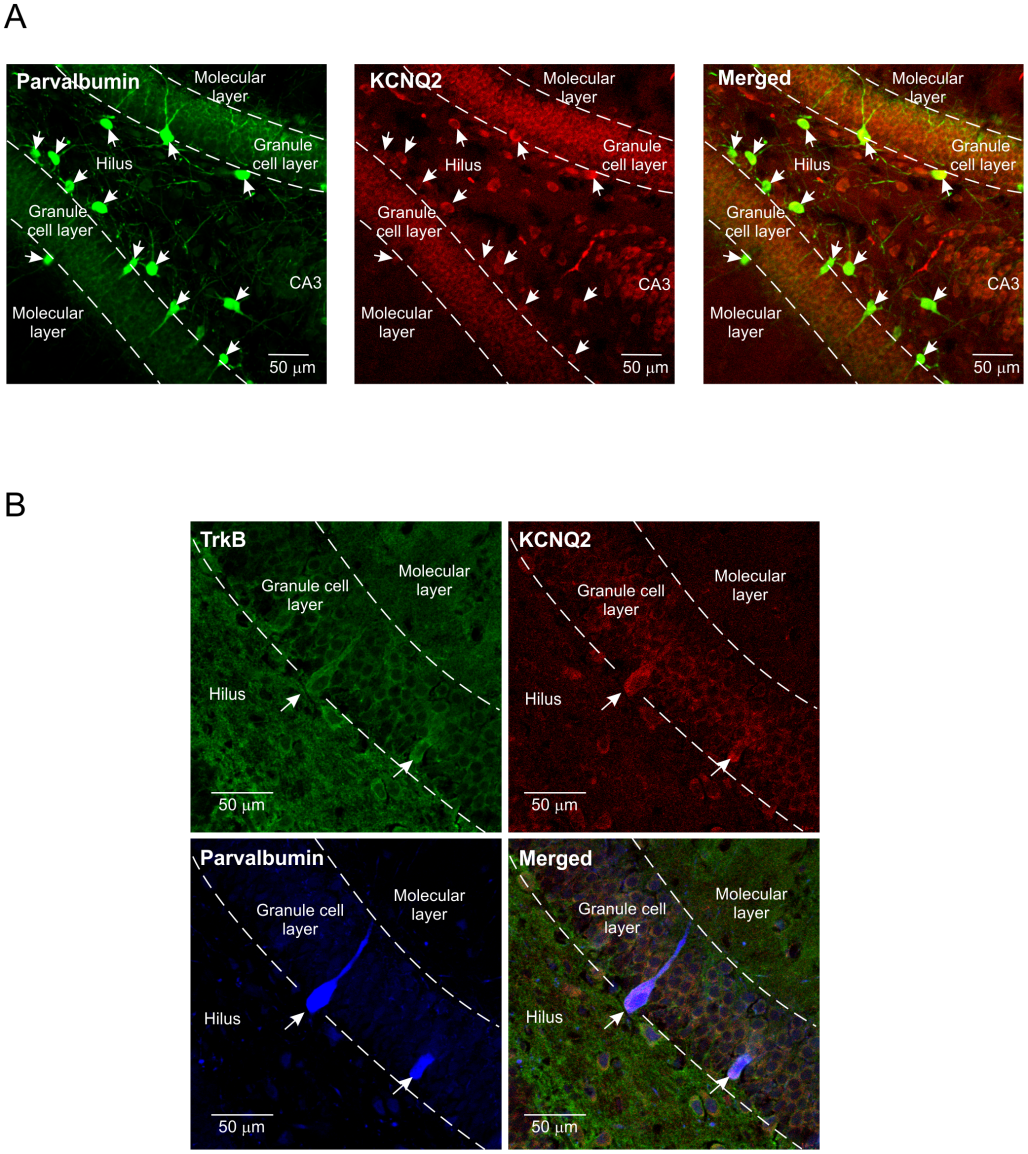
Co-localization of TrkB receptors and Kv7.2/KCNQ2 channels in parvalbumin-positive interneurons in rat dentate gyrus. A. Double immunohistochemistry showing the distribution of parvalbumin-positive interneurons (green) and Kv7.2/KCNQ2 channels (red) in dentate gyrus. B. Triple immunohistochemistry indicating the co-localization of TrkB receptors (green), Kv7.2/KCNQ2 channels (red) and parvalbumin-positive interneurons (blue).

The electrophysiological data pointed to a modulation of I_M_ by BDNF likely through TrkB receptors [7]. To detect M-channels in these interneurons, we performed double immunohistochemistry against parvalbumin and Kv7.2/KCNQ2 channels. All parvalbumin-positive interneurons expressed Kv7.2/KCNQ2 channels (Fig. 5A). While we have previously shown that parvalbumin-positive mouse interneurons express TrkB receptors [7], we went on to confirm that Kv7.2/KCNQ2 channels and TrkB co-localized in parvalbumin-positive rat interneurons, as illustrated by the triple immunohistochemistry in Fig. 5B.

## Discussion

In this work, we studied the mechanism through which BDNF modulates the firing of parvalbumin-positive interneurons in rat dentate gyrus. According to the results, BDNF produced a decrease in the firing rate accompanied by a decrement in the input resistance, with no change in the resting membrane potential or action potential waveform. Also, after application of BDNF, the delay to the first spike observed at near-threshold voltage ranges was abolished. The depressant effect of BDNF on the firing rate was mediated by TEA-sensitive conductances indicating the possibility that potassium currents could be involved, as indeed found by using agonist (retigabine) and antagonist (XE991) of Kv7/KCNQ/M channels in current- and voltage-clamp experiments. Thus, we found that BDNF through TrkB receptors could modulate the firing of interneurons by interacting with G proteins and/or PLC intracellularly. In agreement, parvalbumin-positive interneurons co-expressed TrkB receptors and KCNQ2 channels.

### BNDF influences the GABAergic system

There are several earlier reports that BDNF affects the GABAergic system. Most recently, BDNF can enhance GABA release from parvalbumin-positive interneurons by promoting the differentiation and maturation of adult-born neurons in the subgranular zone of the mouse dentate gyrus [2]. However, more relevant to our findings, and when looking a more acute effects of BDNF, exogenous application depresses GABA release from the terminals in the CA1 region [16] and dentate gyrus [7]. This appears to be the major short-term effect of BDNF, in addition to our current finding that BDNF modulates, on a rapid time scale, the excitability of somata of parvalbumin-positive interneurons of rat dentate gyrus.

### Potential mechanisms underlying BDNF effects on rat dentate gyrus interneurons

BDNF depressed the excitability of interneurons with no significant changes in spike shape or membrane potential, arguing against a modulation of leak channels that prevented repetitive firing. The fact that the spike waveform and half-width were not affected by BDNF suggests that Kv3.2 delayed rectifier channels, which nominally mediate the rapid repolarization of action potentials in parvalbumin basket cells [17] are not modulated by BDNF. This possibility has also been reported in parvalbumin basket cells in relation to muscarinic acetylcholine receptor activation [18].

Bath application of TEA, a general blocker of potassium channels, abolished the depressant effect of BDNF indicating that the effect is mediated by potassium currents. As we argue below, a strong candidate to be modulated by BDNF is Kv7/KCNQ/M channels that are known to play a key role in regulating excitability of central and peripheral neurons (for review see [19], including hippocampal interneurons [8].

### TrkB signaling pathways

Trk receptors are typical receptor tyrosine kinases whose activation is stimulated by neurotrophin-mediated dimerization and autophosphorylation of tyrosine residues in the intracellular kinase domain [20]. Within seconds to minutes, the tyrosine phosphorylation is followed by the activation of diverse signaling cascades, such as the phosphatidylinositol 3-kinase (PI3K)/Akt, MAPK, and PLC-γ pathways and their downstream effectors [5], [21]. Here, we found that the depressant effect of BDNF was mediated by protein G and PLC dependent cascades, indicating that BDNF exerts its modulation through both pathways, but probably not involving global rises in intracellular calcium. We hypothesize that TrkB mediates G protein-dependent PLC activation, which catalyzes the breakdown of lipids to diacylglycerol and inositol(1,4,5)triphosphate (IP3) [22]. Binding of IP3 to specific receptors promotes release of calcium from intracellular stores, while diacylglycerol allows maximal activation of several protein kinase C isoforms that could promote neuronal plasticity as described by others [5], [23].

### BDNF modulates I_M_ in rat dentate gyrus interneurons

To investigate whether I_M_ was modulated by BDNF we performed voltage-clamp experiment to isolate an M-like current that was observed by the typical slow inward deactivation current [9]. Bath application of BDNF produced an increased I_M_ and this effect was mimicked by retigabine and abolished by XE991, the latter accompanied by no changes in the holding current. In contrast, a change in the holding current was observed when retigabine, or XE991 in combination with BDNF, were applied. These results, together with the TEA experiments, lead to two conclusions. Firstly, BDNF modulates an M-like current increasing its amplitude; secondly, additional potassium currents might be suppressed by BDNF compensating for the opening of M-channels and maintaining the resting membrane potential unchanged. It has been reported that fast-spiking GABAergic interneurons exhibit a delay to first spike near-threshold voltage range [15], [24], [25] as we observed in the present work (Fig. 1). This feature has been linked to the presence of Kv1 potassium channels and their blockade converts the delayed-type discharge pattern to one of continuous spiking with no change in firing frequency [15]. We hypothesize that voltage-dependent Kv1 channels that determine the resting membrane potential and regulate excitability of neurons, are also modulated by BDNF [6] in combination with the I_M_ (current data), thereby controlling the firing frequency at near-threshold voltage range.

### Functional implications

Parvalbumin-positive interneurons are preferentially innervated by recurrent glutamatergic mossy fibers [26]. Thus, these interneurons are in a position to provide a strong feedback inhibition to granule cells in the dentate gyrus. Furthermore, distal dendrites of parvalbumin-positive interneurons receive input from the entorhinal cortex [27] providing feed-forward inhibition to granule cells.

In this context, and besides its involvement in neural development and cell survival, BDNF is essential for synaptic plasticity. It is speculated that pathophysiological levels of BDNF (up or downregulation) may contribute to conditions such as epilepsy or neurodegenerative diseases like Alzheimer’s, Parkinson’s and Huntington’s disease and other neuropsychiatric disorders [1], [28]–[30].

As extensively reported, the dentate gyrus is an important structure implicated in seizures, and *in vitro* and *in vivo* studies have shown involvement of BDNF in the development of temporal lobe epilepsy [28], [31]. Most of the evidences linking BDNF to epilepsy are based on the fact that BDNF is markedly upregulated by seizure activity, especially in the dentate gyrus and CA1–CA3 pyramidal cell layers [32]. This upregulation of BDNF potentially increases excitability, and indeed intra-hippocampal infusion of BDNF is sufficient to induce seizure activity *in vivo* [33]. Several mechanisms have been described in epilepsy that could affect GABAergic inhibition including a loss of interneurons [34] or a decrease in the K^+^-Cl^−^ cotransporter KCC2 [35] leading to depolarizing GABA responses. Moreover, it has been reported that TrkB signaling in parvalbumin-positive interneurons is important for gamma-band network synchronization [36] which is affected in epilepsy [37], [38]. Based upon our current data, we propose that BDNF could decrease the excitability of parvalbumin-positive interneurons, which deliver prominent inhibitory input to granule cells, through an M-like current in dentate gyrus, which might increase the excitability of dentate gyrus granule cells.
